# Considerations About the Indirect Role of Low Cortisone in Subjects With Normal Cortisol to Cortisone Ratio

**DOI:** 10.1210/jendso/bvae095

**Published:** 2024-05-23

**Authors:** Decio Armanini, Simona Censi, Luciana Bordin, Alessandra Andrisani, Chiara Sabbadin

**Affiliations:** Department of Medicine (DIMED), University of Padua, 35128 Padua, Italy; Unit of Endocrinology, University Hospital of Padua, 35128 Padua, Italy; Department of Medicine (DIMED), University of Padua, 35128 Padua, Italy; Unit of Endocrinology, University Hospital of Padua, 35128 Padua, Italy; Department of Women's and Children's Health, University of Padua, 35128 Padua, Italy; Department of Women's and Children's Health, University of Padua, 35128 Padua, Italy; Unit of Endocrinology, University Hospital of Padua, 35128 Padua, Italy

**Keywords:** 11β-hydroxysteroid dehydrogenase, aldosterone, cortisol, cortisone, mineralocorticoid receptor, hypertension

Tapia-Castillo and collaborators [1] report that low cortisone (E) can be a predictor of a low-renin phenotype and is associated with high blood pressure and biomarkers of vascular and renal damage. The study also found that aldosterone was associated with an impaired urinary electrolyte concentration and altered renal function. Twenty-seven percent of the subjects with low E had a high cortisol (F) to cortisone ratio (F/E), which suggests an apparent mineralocorticoid excess (AME) due to a partial deficiency of 11β-hydroxysteroid dehydrogenase type 2 (11βHSD2) enzyme or a nonclassical AME phenotype. Thus, low E could also be important and complementary in the screening of primary aldosteronism. The authors found that in some subjects low E was associated with a normal F/E ratio and could be considered as a predictor of low renin activity, inflammation, vascular and renal damage, confirmed by higher albuminuria and plasminogen activator inhibitor-1 (PAI-1) levels and lower sodium excretion, independently of high or normal blood pressure values. The authors also found that subjects with low E were older than those with normal E, suggesting a decrease in 11βHSD2 expression with age as a possible causal mechanism.

The concentration and regulation of F and E in vivo is more complex, involving not only 11βHSD2, but also several other factors: aldosterone, renin, adrenocorticotropin (ACTH), 11βHSD1, steroid-binding proteins, aldosterone synthase (CYP11B2), mineralocorticoid receptors (MRs), and glucocorticoid receptors (GRs). Finally, the amount of NADP and NADPH available determines the direction of the reaction catalyzed by 11βHSD. 11βHSD1 is an NADP^+^-dependent oxidoreductase, usually reductase, of major glucocorticoids. The NAD^+^-dependent 11βHSD2 is an oxidase that inactivates F, conferring extrinsic specificity of the MR for aldosterone. Many studies have shown that MRs are also present in other tissues, such as cardiomyocytes, endothelial cells, inflammatory cells, and mononuclear leukocytes (MNLs), and in these tissues, which do not possess 11βHSD2, the main ligand is F. Lombes reported also that the MR can discriminate aldosterone from glucocorticoids independently of the 11βHSD [2].

The interrelationship of all the cited actors is complex and is regulated also by individual factors. Excess of adipose tissue leads to an increased amount of 11βHSD1, and the activation of F from E is involved in progression of obesity or overweight [3]. It is also known that 11βHSD2, renin, and aldosterone decrease with age [4]. The decrease of aldosterone is also linked to a progressive age-related sclerosis of the juxtaglomerular apparatus in the kidney, leading to lower renin secretion. The subjects with low E studied by Tapia-Castillo [1] have a higher body mass index (BMI) and this can be consistent with an increased activity of 11βHSD1 in fat, increasing F, and decreasing E due to abundance of the enzyme in this tissue [5]. This situation is finally regulated by the feedback mechanism involving F and ACTH, but not E.

Further actors involved inflammatory diseases, such as hypertension, cardiovascular diseases, and obesity, are circulating and infiltrating mononuclear cells, such as lymphocytes, monocytes, and, locally, macrophages dendritic cells, and fibroblasts. In 1985 we characterized MRs in MNLs [6] and later reported that these cells possess only 11βHSD1 [7]. It is known that these cells also possess GRs, and the concentration ratio of these receptors is 30-fold that of MRs. Circulating MNLs can bring the receptors in all tissues and in situations of inflammation their MRs are activated, contributing to cardiovascular, renal, and cerebrovascular diseases [8]. Several studies have shown that MR antagonists protect heart and other tissues from inflammatory complications [8].

Obesity is another inflammatory disease, and it is interesting to note that the study reports increased protein expression of PAI-1 both in subjects with low E and low renin, but both had increased BMI [1]. It would be interesting to evaluate another group of subjects with normal BMI to distinguish the involvement of overweight and 11βHSD1 in this situation.

We previously reported that MNL incubation with high amounts of aldosterone increases protein expression of PAI-1 and p22phox, both markers of inflammation. Local inflammation due to MR activation can aggravate the inflammatory reaction. Another possible explanation is that excess intracellular F in inflammatory cells can saturate 11βHSD1 in nonclassical target tissues for aldosterone, allowing binding of aldosterone to MRs. A problem that needs better investigation is the presence in some tissues of both 11βHSD1 and 11βHSD2 and MRs and GRs, as for instance in the kidney. The invasion of inflammatory circulating mononuclear cells also brings 11βHSD1, which could play a role of response to inflammation activating F, which has an anti-inflammatory role [8]. The anti-inflammatory reaction can become inflammatory when all GRs are saturated, leading to binding of F and aldosterone to MRs. Finally, the presence CYP11B2 in inflammatory cells and in adipocytes can also be considered in the evaluation of patients with inflammatory diseases, leading to an intracellular increase of aldosterone.

From these observations we conclude that both 11βHSD1 and 11βHSD2 are involved in regulation of the plasma concentration of F, E, aldosterone, and renin, but finally the F concentration is regulated by ACTH and corticotropin-releasing hormone. An important factor to be considered is also the involution with age of renin, GRs, and MRs, leading to a relative increase of F [4].

The measurement of plasma and urinary E can be of importance in subjects without an increased F/E ratio, to evaluate the interrelationship between all these factors and to study the implication of increase of BMI, hypertension, local inflammation, autoimmunity, kidney diseases, and aging, as pointed out by the authors. The study of Tapia-Castillo [1] reports that measurement of E, an inert hormone, could have an important role in the evaluation of both normotensive and hypertensive patients who show variations in renin, F, and aldosterone, even within the normal range. Measurement of E in normotensive subjects could be a possible marker of future development of hypertension and the inflammatory state. This topic needs further studies considering the complexity of regulation of these steroids in vivo.

## Disclosures

D.A. is on Editorial Board of the Journal of the Endocrine Society and of Endocrinology; the other authors declare no conflict of interest.

## Abbreviations

11βHSD: 11β-hydroxysteroid dehydrogenase
ACTH: adrenocorticotropin
AME: apparent mineralocorticoid excess
BMI: body mass index
E: cortisone
F: cortisol
F/E: cortisol to cortisone ratio
GR: glucocorticoid receptor
MNL: mononuclear leukocyte
MR: mineralocorticoid receptor
PAI-1: plasminogen activator inhibitor-1
